# Protein-based biofilm matrices in Staphylococci

**DOI:** 10.3389/fcimb.2014.00171

**Published:** 2014-12-10

**Authors:** Pietro Speziale, Giampiero Pietrocola, Timothy J. Foster, Joan A. Geoghegan

**Affiliations:** ^1^Department of Molecular Medicine, Unit of Biochemistry, University of PaviaPavia, Italy; ^2^Department of Microbiology, Moyne Institute of Preventive Medicine, Trinity College DublinDublin, Ireland

**Keywords:** *Staphylococcus*, biofilm, cell wall-anchored proteins, extracellular proteins, homophilic interactions

## Abstract

*Staphylococcus aureus* and *Staphylococcus epidermidis* are the most important etiological agents of biofilm associated-infections on indwelling medical devices. Biofilm infections may also develop independently of indwelling devices, e.g., in native valve endocarditis, bone tissue, and open wounds. After attachment to tissue or indwelling medical devices that have been conditioned with host plasma proteins, staphylococcal biofilms grow, and produce a specific environment which provides the conditions for cell–cell interaction and formation of multicellular communities. Bacteria living in biofilms express a variety of macromolecules, including exopolysaccharides, proteins, extracellular eDNA, and other polymers. The *S. aureus* surface protein C and G (SasC and SasG), clumping factor B (ClfB), serine aspartate repeat protein (SdrC), the biofilm-associated protein (Bap), and the fibronectin/fibrinogen-binding proteins (FnBPA and FnBPB) are individually implicated in biofilm matrix formation. In *S. epidermidis*, a protein named accumulation-associated protein (Aap) contributes to both the primary attachment phase and the establishment of intercellular connections by forming fibrils on the cell surface. In *S. epidermidis*, proteinaceous biofilm formation can also be mediated by the extracellular matrix binding protein (Embp) and *S. epidermidis* surface protein C (SesC). Additionally, multifunctional proteins such as extracellular adherence protein (Eap) and extracellular matrix protein binding protein (Emp) of *S. aureus* and the iron-regulated surface determinant protein C (IsdC) of *S. lugdunensis* can promote biofilm formation in iron-depleted conditions. This multitude of proteins intervene at different stages of biofilm formation with certain proteins contributing to biofilm accumulation and others mediating primary attachment to surfaces. This review examines the contribution of proteins to biofilm formation in Staphylococci. The potential to develop vaccines to prevent protein-dependent biofilm formation during staphylococcal infection is discussed.

## Introduction

**Figure 1:**
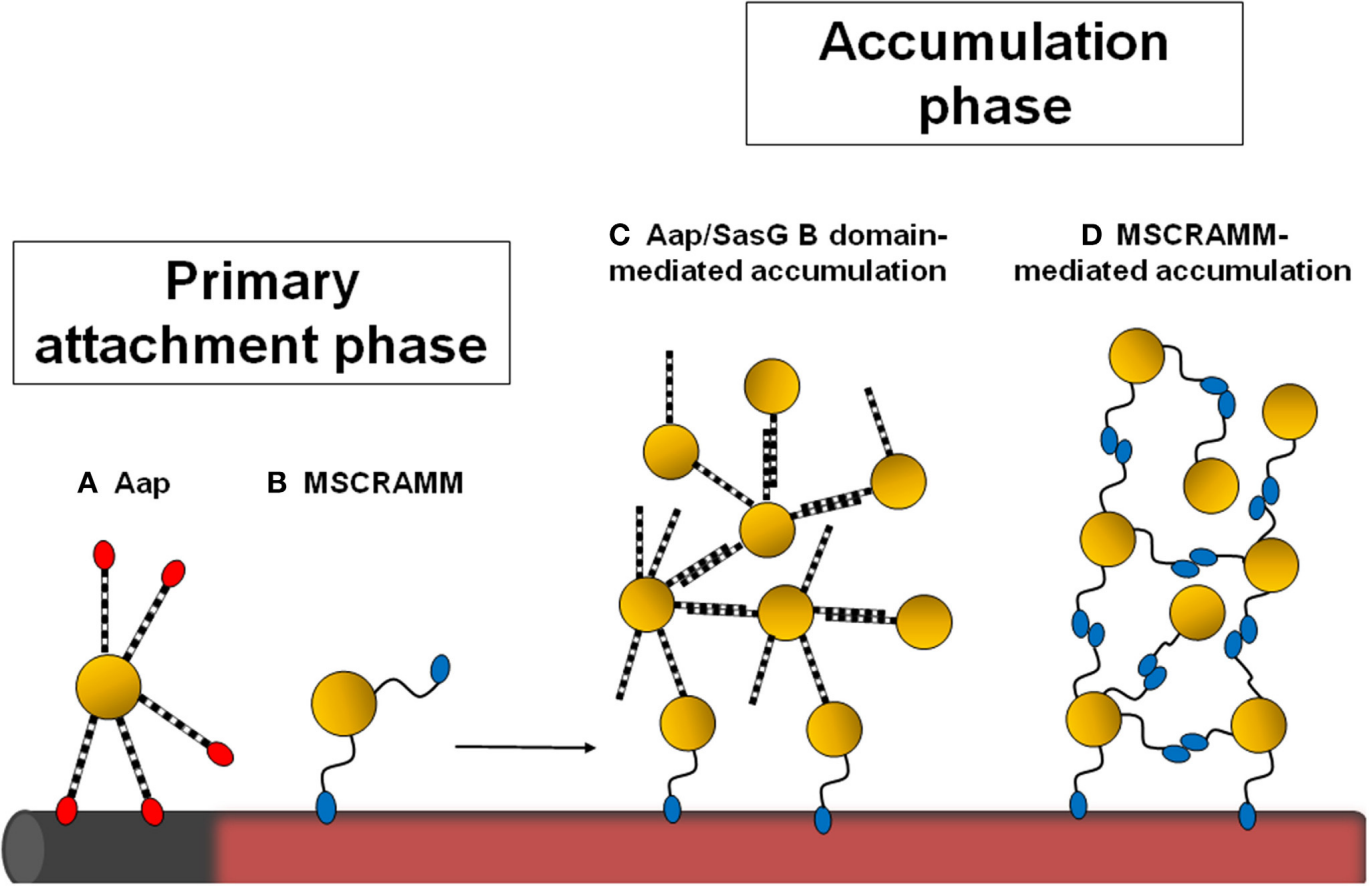
**Schematic diagram showing the stages of staphylococcal biofilm formation influenced by cell wall-anchored proteins**. Staphylococci can attach to the naked surface of a foreign device (shown in gray) or to a device that has become coated with host plasma components (pink). The Aap A domain (in red) promotes primary attachment to uncoated surfaces **(A)**. Attachment to plasma-coated surfaces is mediated by MSCRAMMs **(B)**. If the Aap/SasG A domain is removed by proteolytic cleavage, the B region can promote intercellular accumulation **(C)**. Alternatively, homophilic interactions between staphylococcal MSCRAMMs on different cells mediate biofilm accumulation **(D)**.

*Staphylococcus aureus* and *Staphylococcus epidermidis* cause a broad spectrum of diseases in humans ranging from soft tissue infections and abscesses in organ tissues to osteomyelitis, endocarditis, and toxic shock syndrome. It is not surprising that these bacteria, especially *S. aureus*, encode a large array of virulence factors that enable the organisms to infect different tissues within the host. Both species display a strong capacity to form biofilms, which are functional multilayered communities of microrganisms adhering to a surface embedded in a self-synthesized extracellular matrix. Biofilm infections are important clinically because bacteria in biofilms exhibit recalcitrance to antimicrobial compounds and persistence in spite of sustained host defenses. The development of a bacterial biofilm is a complex, multifactorial process and can be divided into three phases which involve specific molecular factors: attachment, accumulation/maturation, and detachment/dispersal (O'Toole et al., 2000; Otto, 2013). Initial attachment can occur on inert or biotic surfaces. Attachment of Staphylococci to an abiotic surface, such as the naked plastic or metal surface of an indwelling medical device, is dependent on the physico-chemical characteristics of the device and bacterial surface components such as the accumulation-associated protein (Aap) (Conlon et al., 2014), autolysins AtlA (Houston et al., 2011; Bose et al., 2012) and AtlE (Rupp et al., 2001) or wall teichoic (WTA) and lipoteichoic acids (LTA) (Gross et al., 2001). Primary attachment to a biotic surface in host tissues and synthetic surfaces coated with plasma proteins, such as fibronectin, fibrinogen, and vitronectin, is governed by cell wall-anchored (CWA) proteins including clumping factors A and B and the fibrinogen/fibronectin-binding proteins FnBPA and FnBPB from *S. aureus* or the fibrinogen-binding protein SdrG/Fbe from *S. epidermidis* (Vaudaux et al., 1995). Once attachment to tissue or matrix-covered devices is accomplished, staphylococcal biofilms grow by proliferation and production of a scaffolding extracellular matrix. Until recently the only known matrix components were polysaccharide intercellular adhesin (PIA), also known as poly-N-acetyl-glucosamine (PNAG) (Mack et al., 1996), and extracellular DNA (eDNA) (Montanaro et al., 2011). PIA, which has a net positive charge, may promote intercellular interactions by binding to the negatively charged surfaces of bacterial cells. It is now recognized that several staphylococcal surface proteins can also promote the accumulation phase in an *ica*-independent manner (Foster et al., 2014). Thus, CWA proteins mediate primary attachment and also promote intercellular adhesion and biofilm accumulation and maturation (Figure 1). This is followed by the dispersal phase where the biofilm structure is disrupted by enzymatic degradation of matrix components, most notably by proteases (Boles and Horswill, 2008), nucleases (Sharma-Kuinkel et al., 2009; Kiedrowski et al., 2011; Beenken et al., 2012), and a group of small amphiphilic α-helical peptides, known as phenol-soluble modulins (PSMs) functioning as surfactants (Wang et al., 2011; Periasamy et al., 2012).

This review will focus on the role of surface proteins in biofilm formation, with particular emphasis on the recent discoveries that several CWA proteins promote accumulation by specific homophilic interactions.

## CWA proteins

**Figure 2:**
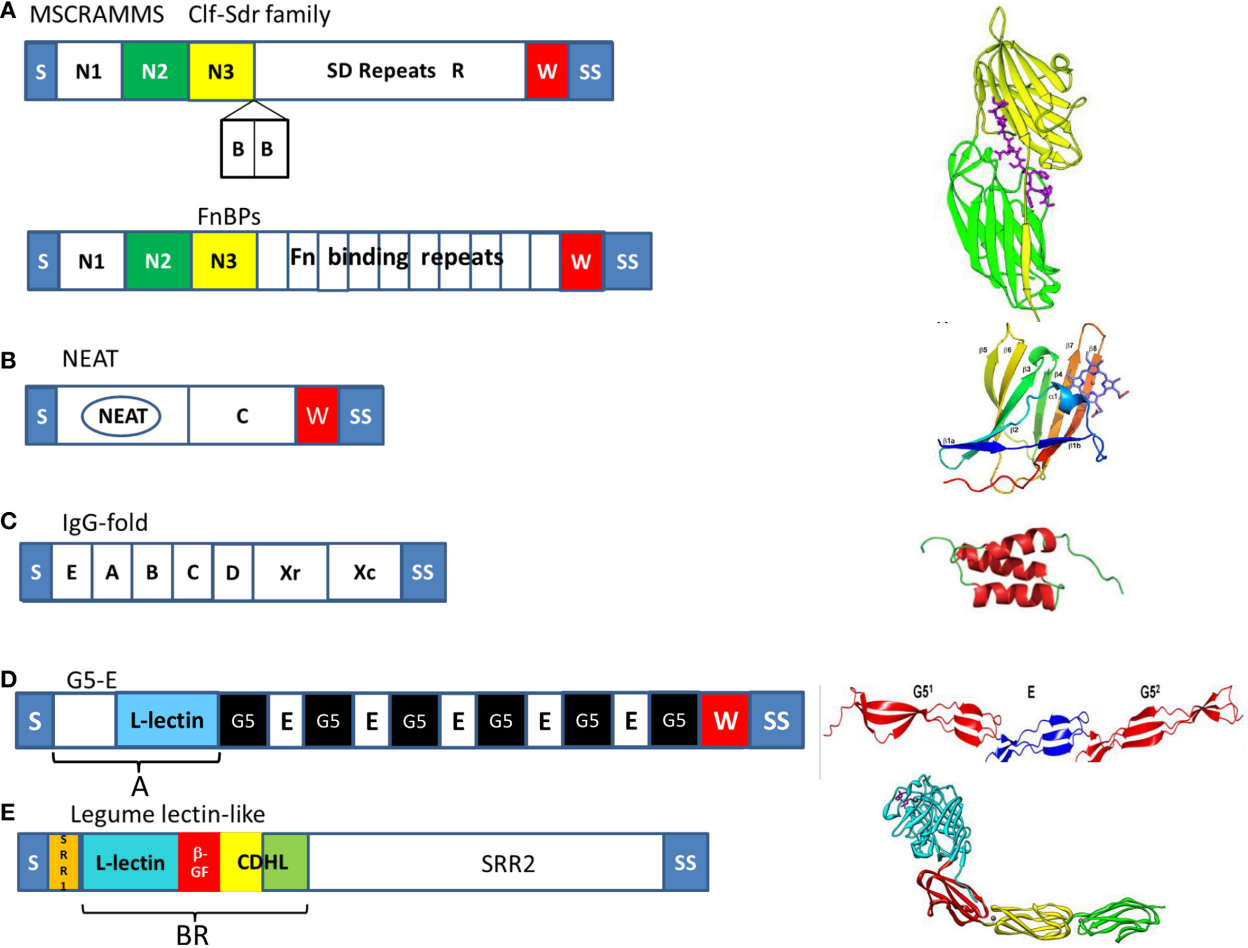
**Organization of the major families of cell wall-anchored proteins of Staphylococci**. The diagrams to the left show the organization of the proteins into subdomains and on the right the structure of the defining subdomain; tandemly arrayed IgG-like folds N2 (green) and N3 (yellow) of MSCRAMMs, a NEAT motif in Isd proteins, triple helical bundles in protein A, G5-E repeats in Aap and SasG, and the BR domain of SraP comprising a legume-like lectin domain (cyan), a β-grasp fold domain (β-GF, red), and two cadherin-like domains (CDHL, yellow and green). Common features of CWA proteins are S, secretory signal sequence, W, wall spanning region and SS, the sorting signal.

The surfaces of staphylococcal cells are decorated with a variety of CWA proteins that are anchored to peptidoglycan by the enzymatic activity of sortases (Foster et al., 2014) (Figure 2). The precise repertoire of CWA proteins varies among strains. *S. aureus* can express up to 24 different CWA proteins whereas coagulase-negative Staphylococci such as *S. epidermidis* and *S. lugdunensis* express a smaller number. Moreover, the expression of CWA proteins can be altered by growth conditions. For example, some proteins are expressed only under iron-limited conditions, whereas others are found predominantly on cells in the exponential or stationary phases of growth.

Secretory signal sequences that are located at the amino termini direct the translated proteins to the secretory (Sec) apparatus in the membrane and are cleaved during secretion. At their carboxyl termini, each of these proteins has a characteristic sorting signal, which facilitates their covalent anchorage to peptidoglycan. The housekeeping sortase A anchors the majority of CWA proteins which have the LPXTG motif within their sorting signal. In contrast, sortase B of *S. aureus* and *S. lugdunensis* anchors Isd proteins which have sorting signals with the motif NPQxN/P and which are only expressed under iron-restricted conditions (Foster et al., 2014).

It has been proposed recently (Foster et al., 2014) that CWA proteins be classified primarily based on structural and functional considerations (Figure 2). The microbial surface component recognizing adhesive matrix molecules (MSCRAMM) family comprises proteins with tandemly-linked IgG-like folds in the N-terminal A region. In the archetypal MSCRAMMs SdrG, ClfA, and ClfB the N2 and N3 subdomains are sufficient to promote binding to ligands by the dock, lock, and latch (DLL) mechanism. Linking the A region to the cell wall-anchoring domain are serine-aspartate dipeptide repeats of varying length in the case of the Clf–Sdr subfamily, or tandem repeats of fibronectin binding domain in the case of FnBPs. The Sdr proteins have additional 110–113 residue B repeats located between the A region and the SD repeat region that act as rigid rods to project the A domain further from the cell surface (Foster et al., 2014).

Near iron transporter (NEAT) motif proteins are involved in heme capture from hemoglobin and help bacteria to survive in the host, where iron is restricted. The defining characteristic of Isd CWA proteins is the presence of one or more NEAT motifs, which bind either hemoglobin or heme (Hammer and Skaar, 2011). The CWA Isd proteins also have functions other than those involved in heme transport.

Protein A is a multifunctional CWA protein that is ubiquitous in *S. aureus*. At the N terminus, protein A contains four or five homologous modules (known as EABCD), each of which consists of single separately folded three-helical bundles that can bind to several distinct ligands. Located between this region and the cell surface is region Xr, which is composed of octapeptide repeats that are highly variable in number, followed by a constant region Xc (Foster et al., 2014).

The serine-rich adhesin for platelets SraP has a complex N-terminal domain comprising short serine-rich repeats followed by a B region (BR) that is subdivided into four subdomains, a legume-like (L-type) lectin domain that is responsible for adhesion to glycoproteins containing N-acetyl neuraminic acid, a β-grasp fold domain and two cadherin-like domains (Yang et al., 2014).

*S. aureus* surface protein G (SasG) is closely related to Aap of *S. epidermidis*. Both proteins have repeated G5 domains separated by 50-residue sequences known as E regions (Gruszka et al., 2012; Conrady et al., 2013). The G5-E domains of SasG and Aap share 64% amino-acid identity. At the N-termini of the proteins are N-terminal A domains (Roche et al., 2003). Within the A domains of SasG and Aap is a L-type lectin domain.

## Evidence for the involvement of CWA proteins in biofilm

### Identification

The first step in investigating an unknown mechanism of biofilm formation is to determine if the matrix is composed of protein and/or polysaccharide by incubating an established biofilm with a protease such as trypsin or with periodate which oxidizes glucose-containing polysaccharides. A reduction in the integrity of the biofilm by protease treatment is a clear indication of the involvement of protein. The absence of the *ica* genes required for biosynthesis of the PNAG/PIA and/or a lack of detectable extracellular polysaccharide on the cell surface is consistent with a novel, perhaps protein-dependent, mechanism.

The morphology of cells visualized by scanning electron microscopy in a PIA/PNAG biofilm matrix is quite distinct from biofilm involving proteins. In the former, cells are embedded in copious extracellular material while cells from a protein-dependent biofilm are in close contact without a detectable extracellular matrix (Vergara-Irigaray et al., 2009).

Site-specific mutagenesis offers a clear-cut method for determining if a CWA protein is involved. Loss of biofilm in a null mutant defective in sortase A or sortase B suggests the involvement of a CWA protein. Systematic inactivation of genes encoding individual CWA proteins will identify the individual component(s). A mutant defective in a single CWA protein might not give a completely defective phenotype because two or more proteins might contribute. For example, in the case of FnBP-dependent biofilm, inactivation of both FnBPA and FnBPB was required to eliminate biofilm formation completely (O'Neill et al., 2008; Vergara-Irigaray et al., 2009).

Transposon mutagenesis followed by identification of the inactivated gene showed that the Bap protein was involved in biofilm formation by *S. aureus* bovine mastitis strain V329 (Cucarella et al., 2001). This approach could also suggest a role for non-covalently anchored proteins as well as identify potential regulators controlling expression of biofilm-associated proteins.

Once a CWA protein has been identified by mutagenesis or is suspected from other evidence, the gene can be cloned into a plasmid vector and used to complement the mutation or to express the protein in a surrogate host, either a different strain of the same species that naturally lacks the gene in question or in a heterologous host such as *S. carnosus* or *Lactococcus lactis*. If the gene is placed under the control of a regulatable promoter then the concentration of the inducer can be used to control the density of biofilm.

Many CWA proteins are composed of several distinct domains (Figure 2). Further genetic manipulation can help identify the domain involved in biofilm. Staphylococcal cells expressing SasG B repeats but not the A domains still formed biofilm whereas cells expressing A domains but lacking B repeats did not (Geoghegan et al., 2010). This strongly implicated the B repeats in biofilm formation. Subdomain N2 of the A region of SdrC was also implicated by a similar approach (Barbu et al., 2014).

Individual subdomains can be cloned and expressed as recombinant proteins. Inhibition of biofilm formation by incubation of the growing culture with purified recombinant proteins provided evidence for the role of the B domains of SasG/Aap and the N2 region of SdrC (Geoghegan et al., 2010; Barbu et al., 2014). Antibodies raised against individual subdomains have also been used to inhibit biofilm formation and to support studies with recombinant proteins and expression of truncates.

### Homophilic interactions

Specific homophilic interactions between CWA proteins expressed on different cells are likely to be an important mechanism of cell–cell accumulation during biofilm development (Figure 1). The ability of purified recombinant CWA protein to bind to bacterial cells expressing the protein on their surface provided preliminary evidence for homophilic interactions for SraP (Sanchez et al., 2010), IsdC (Missineo et al., 2014), and SasG (Geoghegan et al., 2010) mediated biofilm accumulation.

If the CWA proteins can engage in homophilic interactions during biofilm accumulation, purified recombinant proteins should be able to form dimers in solution. This is certainly the case for Aap/SasG, IsdC, SraP, and SdrC (Conrady et al., 2008; Geoghegan et al., 2010; Missineo et al., 2014; Yang et al., 2014).

**Figure 3:**
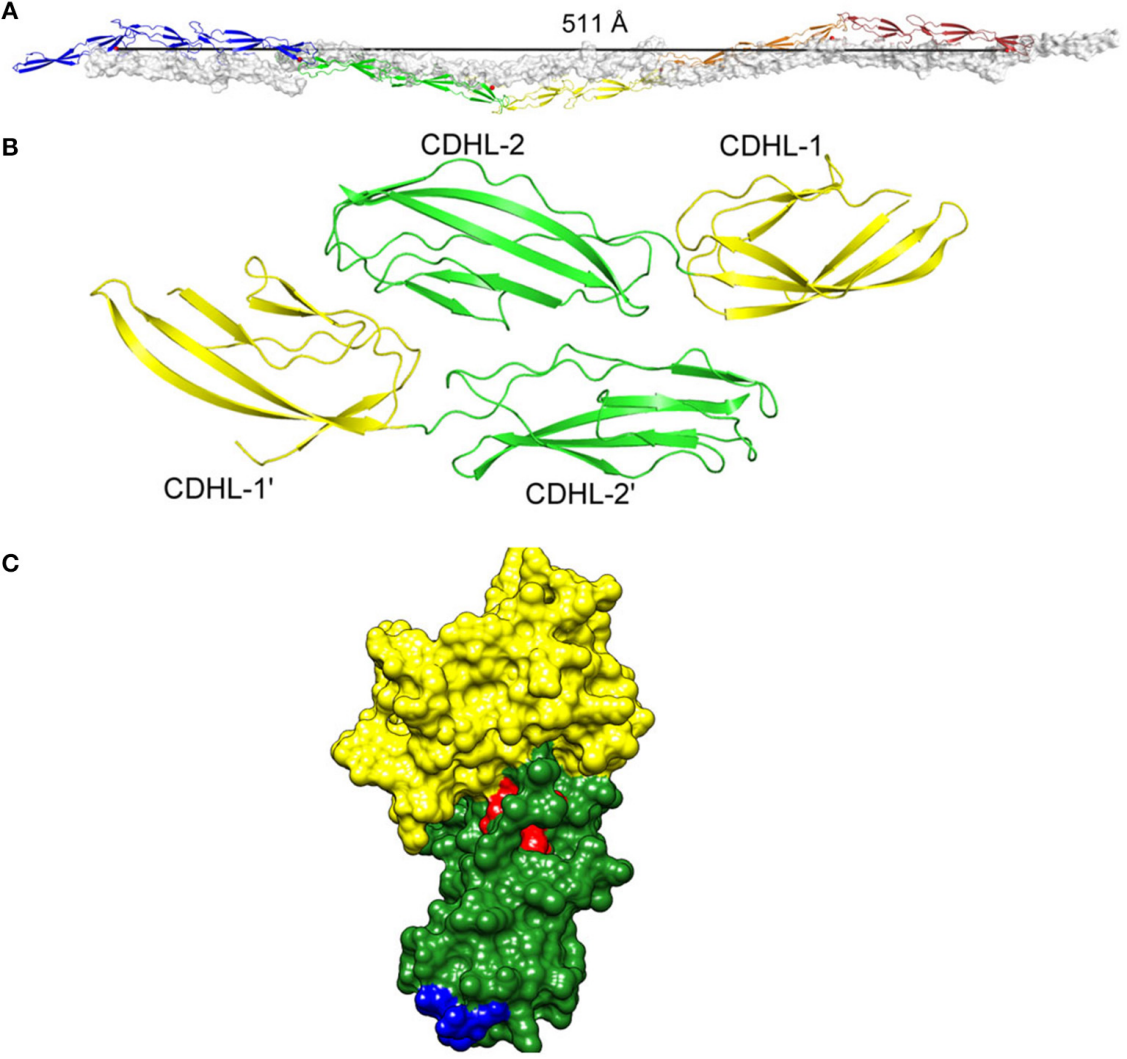
**(A)** Homophilic interactions between cell wall-anchored proteins. Five repeated G5-E domains of Aap form an anti-parallel twisted rope. **(B)** The SraP cadherin-like domain dimer. **(C)** The N2 (green) and N3 (yellow) subdomains of the A region of SdrC modeled on the crystal structure of ClfA with the residues involved in forming the homophilic interaction domains shown in red and blue. Figure adapted from Conrady et al. (2013).

Phage display screening first identified the putative interaction domains within the N2 subdomain of SdrC (Barbu et al., 2014). M13 phages expressing a random 12 amino acid peptide library were panned against the recombinant SdrC A domain and two consensus peptides within subdomain N2 were identified (Barbu et al., 2014) (Figure 3).

Proof of the mechanism of homophilic binding and identification of residues involved will be provided by the X-ray crystal structure of the dimers formed in solution. This has been achieved with the cadherin-like domains of SraP (Yang et al., 2014) and the G5-E (B repeats) of Aap (Conrady et al., 2013) (Figure 3).

### AaP/SasG

The Aap and SasG proteins of *S. epidermidis* and *S. aureus*, respectively, have very similar structural and functional organization. Aap can promote either the primary attachment or accumulation phase of biofilm formation depending on the strain being studied (Figure 1). Primary attachment is mediated by the N-terminal A domain (Conlon et al., 2014) while the B regions mediate biofilm accumulation (Rohde et al., 2005). The A region must be removed by proteolysis (Aap) or by limited digestion within the B repeat region (SasG) to allow the B domains to interact and promote biofilm accumulation (Rohde et al., 2005; Geoghegan et al., 2010). The proteins have 5–17 B repeats, each comprising nearly identical 78 residue G5 subdomains followed by an E spacer region of 50 residues (Figure 2). G5 domains are characterized by five conserved glycine residues, and they adopt a β–triple helix–β-like fold. In general, proteins that comprise highly similar domains in a tandem arrangement are prone to misfolding. As the amino acid sequence of each G5 domain is identical, it is thought that alternating individually folded G5 and E regions is a mechanism to prevent protein misfolding (Gruszka et al., 2012).

The G5-E repeats can undergo a Zn^2+^-dependent homophilic interaction to form an antiparallel twisted cable (Figure 3). The structural basis was studied using short repeat segments but molecular modeling indicated that five repeats are required for complete twists to form (Conrady et al., 2013) (Figure 3). This is consistent with the finding that biofilm formation by SasG requires at least five repeats to be expressed on the cell surface (Corrigan et al., 2007).

The *S. epidermidis* strain CSF41498 requires Aap for primary attachment to surfaces (Conlon et al., 2014). In strain CSF41498, the A domain is not processed by proteases. Thus, Aap is capable of promoting either the primary attachment or accumulation phase of biofilm formation depending on whether the A domain has been removed by proteases (Conlon et al., 2014; Schaeffer et al., 2014).

### SraP

The N-terminal BR domain of SraP forms a rigid rod-like structure that projects the lectin-binding region away from the cell surface (Yang et al., 2014). As well as promoting adhesion to neuraminic acid-containing glycoproteins, the BR region is predicted to promote biofilm formation by a homophilic interaction between the pairs of cadherin-like domains. The crystal structure of dimers showed that CDHL-2 binds across the junction of the tandemly arrayed CDHL-1 and -2 (Yang et al., 2014) (Figure 3).

### FnBPs, SdrC, and ClfB

Several MSCRAMM proteins can promote biofilm accumulation. In each case the N-terminal A domain is responsible. For FnBPs the binding region was further localized to the N2N3 subdomains (Geoghegan et al., 2013), and in the case of SdrC, subdomain N2 (Barbu et al., 2014). The N2N3 subdomains engage in binding to ligands by the DLL mechanism. However, DLL is not involved in FnBPA-mediated biofilm because a strain expressing a variant of FnBPA lacking the latching peptide still formed biofilm (Geoghegan et al., 2013). Furthermore, an asparagine residue in the FnBPA ligand binding trench that is crucial for ligand binding by DLL could be substituted without reducing biofilm formation (Geoghegan et al., 2013).

In certain strains of *S. aureus* growing in Ca^2+^-depleted conditions biofilm formation depends upon ClfB. This is unique in that other biofilm mediators are inhibited by Ca^2+^ depletion (Abraham and Jefferson, 2012). SasG, Aap, and FnBP-dependent biofilm formation requires Zn^2+^ (Conrady et al., 2008; Geoghegan et al., 2010, 2013). In contrast, Mn^2+^ inhibits SdrC dimerization and SdrC-dependent biofilm formation (Barbu et al., 2014).

During biofilm growth, expression of genes encoding a number of CWA proteins, including ClfB and FnBPA, is increased (Resch et al., 2005). It is possible that expression of biofilm-associated genes is influenced differently by regulatory factors in clinical isolates from biofilm-associated infection and laboratory strains of *S. aureus*. For example, HA-MRSA strains are frequently genotypically or phenotypically *agr* negative (Fowler et al., 2004; Rudkin et al., 2012). In addition it is likely that proteases modulate surface protein-dependent biofilm formation. ClfB-mediated biofilm formation has been demonstrated for strains where the aureolysin-encoding gene is inactivated so it is possible that this phenotype is restricted to strains harboring loss of function mutations in the *aur* gene (Abraham and Jefferson, 2012).

### Biological significance

Flow cell systems are a superior method for analysing the contribution of proteins to biofilm formation *in vitro* compared to using static microtiter plate assays. The development of biofilm can be monitored over time. The importance of FnBPs in biofilm formation by HA- and CA-MRSA (O'Neill et al., 2008; Vergara-Irigaray et al., 2009; McCourt et al., 2014) and Aap in biofilm formation by *S. epidermidis* (Conlon et al., 2014; Schaeffer et al., 2014) has been demonstrated using flow cells. It will be important to determine if other proteins can support biofilm formation under flow conditions which more closely mimic the *in vivo* situation.

In the case of FnBPs and Aap, the importance of protein-dependent biofilm formation has been proven *in vivo*. FnBP-deficient mutants colonized catheters implanted in mice poorly while the absence of the *ica* operon had no effect (Vergara-Irigaray et al., 2009). Aap-deficient mutants colonized indwelling catheters less well than the wild-type strain in a rat model of catheter-related infection (Schaeffer et al., 2014). It will be important to test mutants deficient in other factors implicated in protein-dependent biofilm in animal models of foreign body infection.

### Other CWA proteins involved in staphylococcal biofilm formation

This section reviews other CWA proteins with reported roles in biofilm formation but where the mechanistic basis is less well understood (summarized in Table 1). The first surface protein identified that could induce biofilm development was Bap of *S. aureus*. Bap is a large protein of 2276 amino acids whose core region consists of 13 identical tandem repeats of 86 residues. Bap promotes biofilm formation in staphylococcal strains isolated from mammary glands in ruminants suffering from mastitis (Cucarella et al., 2001; Arrizubieta et al., 2004).

**Table 1:** **Surface proteins implicated in staphyococcal biofilm formation**.

**Surface protein**	**Biofilm static^a^**	**Biofilm flow^b^**	**Strains**	**Mutant^c^**	**Expression in surrogate host^d^**	**Over-expression^e^**	**Primary attachment^f^**	**Accumulation^g^**	**Homophilic interaction^h^**
*S. aureus* FnBPs	+	+	MRSA 123 HA-MRSA CC22, CC8 USA300 LAC	+	+ SA		+	+	(+)
SdrC	+	NT	Newman	+	+ LL		+	+	+
SasG	+	NT	8325-4, SH1000		+ SA	+	−	+	(+)
SasC	+	NT			+ SC	+	+	+	NT
ClfB	+	NT	10833 Newman	+	+ LL		NT	+	NT
Spa	+	+				+	NT	+	NT
SasX	+	NT	ST239	+	NT		+	+	NT
SraP	+	NT	ISP479C	+	NT		NT	+	+
Bap	+	+	Bovine V329	+	+ SA		+	+	NT
*S. epidermidis*									
Aap	+	NT	RP62a 5179	+	+ SE		NT	+	+
Embp^*^	+	NT	1585 1457	+	NT	+	+ via Fn	+	NT
*S. lugdunensis*									
IsdC	+	NT	N920143	+	+ LL		+	+	+

NT, not tested.

a Biofilm formation in a standard microtiter dish format with bacteria grown statically.

b Biofilm formation measured under flow conditions.

c The role of the surface protein was demonstrated by studying isogenic mutants.

d The role of the surface protein was demonstrated by expression of the cloned gene in a surrogate host (SA, S. aureus; LL, L. lactis; SC, S. carnosus; SE, S. epidermidis).

e Over-expression of the protein using a multicopy plasmid in a surrogate host, or from the chromosomal gene in a host strain with regulatory mutations leading to high level expression.

f Primary attachment to unconditioned plastic surfaces [or following conditioning with fibronectin (Fn)].

g Accumulation phase measured.

h Homophilic interaction proven, or (in parenthesis) strongly suspected.

* Embp is not sortase-anchored. It lacks a consensus C-terminal sorting signal and is removed from cells by boiling or sonication.

Embp, a giant protein located in the cell wall of *S. epidermidis* and with potential functional similarity to large homologous proteins produced by other pathogenic bacteria such as *S. aureus*, mediates binding of *S. epidermidis* to surface attached fibronectin so is likely to constitute the first step of biofilm formation on conditioned surfaces. The Found In Various Architectures (FIVAR) region of Embp mediates binding of *S. epidermidis* to surface attached fibronectin, while the binding site in fibronectin for Embp was assigned to the fibronectin domain type III12 (Christner et al., 2010).

SasC represents another *S. aureus* CWA protein factor that is involved in cell aggregation and biofilm formation. Expression of full-length SasC or its N-terminal domain, which contains a FIVAR motif, mediates the formation of bacterial aggregates, increased attachment to polystyrene, and enhanced biofilm formation (Schroeder et al., 2009).

Overproduction of protein A by *S. aureus* was shown to be responsible for bacterial aggregation and biofilm formation (Merino et al., 2009). Moreover, exogenous addition of synthetic protein A or bacterial supernatants containing protein A can also promote biofilm development. Protein A-mediated biofilm formation was inhibited by addition of serum or immune IgG (Merino et al., 2009). However, it should be noted that the ability of protein A to promote biofilm formation was only ever demonstrated in a laboratory strain engineered to over express the protein.

A recent study of strain ST239, a dominant MRSA strain in the Far East, showed that expression of SasX, a protein that confers virulence in mouse skin and lung infection, may cause bacterial aggregation and promote biofilm formation (Li et al., 2012).

It is unclear whether aggregation and biofilm formation by these different CWA proteins is the result of homophilic interactions between two identical molecules expressed on the surface of neighboring cells. It is possible that these proteins mediate heterophilic interactions with other surface proteins or with non-proteinaceous cell wall structures.

### Cytoplasmic and secreted proteins also contribute to biofilm formation

Although several CWA proteins of Staphylococci have been identified as important components of the biofilm, the composition of the extracellular matrix still remains uncertain. Recently, it has been reported that the biofilm matrix is largely composed of cytoplasmic proteins that reversibly associate with the cell surface in response to decreasing pH during biofilm formation (Foulston et al., 2014). Additionally, proteins present in the secretome contribute to the composition and formation of staphylococcal biofilm. Eap and Emp are secreted proteins which are non-covalently attached to the *S. aureus* cell surface and have previously been implicated in a number of aspects of *S. aureus* pathogenesis (Chavakis et al., 2005). They are involved in biofilm formation under low-iron growth conditions (Johnson et al., 2008).

The unprocessed wall-anchored major autolysin Atl of *S. aureus* facilitates initial attachment to surfaces in the early events of the FnBP-dependent biofilm phenotype (Houston et al., 2011). Proteolytical cleavage of Atl to the amidase and glucosaminidase domains leads to cell lysis, eDNA release, and cell accumulation. Following these early biofilm events, the FnBPs are required for biofilm maturation (Houston et al., 2011).

Alpha-toxin is a secreted haemolytic toxin which plays an integral role in *S. aureus* biofilm formation. An *hla* mutant was unable to colonize plastic surfaces fully under both static and flow conditions. Thus, it has been proposed that that α-toxin plays a role primarily in cell–cell interactions during biofilm formation (Caiazza and O'Toole, 2003) although the mechanistic basis of this is unclear.

The β-toxin is a neutral sphingomyelinase that lyses erythrocytes and kills proliferating human lymphocytes. It plays a key role in the establishment of *S. aureus* biofilms. This toxin forms covalent cross-links to itself in the presence of DNA, producing an insoluble nucleoprotein matrix *in vitro*, and strongly stimulates biofilm formation *in vivo* as demonstrated by a role in causation of infectious endocarditis in a rabbit model (Huseby et al., 2010).

Amyloid fibrils can also form part of an *S. aureus* biofilm. The PSMs can in certain conditions aggregate to form amyloid fibrils on the surface of the bacterium (Schwartz et al., 2012). Similarly, the signal peptide of the *S. aureus* quorum-sensing molecule AgrD forms amyloid-like aggregates (Schwartz et al., 2014). These fibril structures contribute to overall stability of the biofilm.

### Prevention of biofilm formation by antibodies against CWA proteins

Targeting those processes that occur early in biofilm development and dispersal represents an attractive strategy to interfere with biofilm formation. Considering that many CWA proteins generate a potent immune response, the use of staphylococcal surface-exposed proteins as vaccines represents a promising way to eradicate biofilm formation both *in vitro* and *in vivo*. Several studies have been performed to investigate whether immunization with CWA protein domains can induce protection against biofilm development.

Polyclonal (Rohde et al., 2005) and monoclonal antibodies (Hu et al., 2011) specific to Aap inhibited biofilm formation by strains that develop an Aap-dependent biofilm. Similar inhibitory effects on FnBP-promoted biofilm formation were observed when incubating MRSA strains with Fab fragments recognizing region A of FnBPA (O'Neill et al., 2008). Active vaccination with a recombinant truncated SesC inhibited *S. epidermidis* biofilm formation in a rat model of subcutaneous foreign body infection. Moreover, antibodies to SesC were shown to be opsonic by an *in vitro* opsonophagocytosis assay (Shahrooei et al., 2012). Polyclonal antibodies targeting the phosphonate ABC transporter substrate binding protein (PhnD) inhibited both *S. epidermidis* and *S. aureus* biofilms (Lam et al., 2014). PhnD-specific antibodies blocked biofilm development at the initial attachment and aggregation stages and also served to enhance neutrophil binding, motility, and biofilm engulfment, supporting the concept that PhnD may be a good target for biofilm intervention strategies performed either by vaccination or through passive administration of antibodies (Lam et al., 2014). PSMs have surfactant-like characteristics and the soluble peptides participate in structuring/destructuring biofilms (Wang et al., 2011; Periasamy et al., 2012). In contrast to this, fibrils comprising PSM aggregates preserve the integrity of the biofilm (Schwartz et al., 2012). Antibodies against PSM peptides inhibited bacterial spread from indwelling medical devices suggesting that interference with biofilm detachment mechanisms may prevent dissemination of biofilm-associated infections (Wang et al., 2011).

Brady et al. (2011) identified immunogenic cell wall proteins expressed during an *S. aureus* biofilm infection and used a quadrivalent vaccine, including four of the identified antigens (glucosaminidase, an ABC transporter lipoprotein, a conserved hypothetical protein, and a conserved lipoprotein) combined with antibiotic therapy and demonstrated reduced *S. aureus* biofilm formation on infected tibias using a chronic osteomyelitis model (Brady et al., 2011).

Finally, Gil et al. (2014) found that a common core of secreted proteins (exoproteome) is contained in both exopolysaccharide-based and protein-based biofilm matrices. Intradermal administration of an exoproteome extract of an exopolysaccharide-dependent biofilm induced a humoral immune response and reduced the number of bacterial cells inside a biofilm and on the surrounding tissue using an *in vivo* model of mesh-associated biofilm infection (Gil et al., 2014).

Altogether, these studies demonstrate the potential of biofilm matrix exoproteins and CWA proteins as promising targets for antibody-mediated strategies against staphylococcal biofilm formation.

## Conclusions

Several staphylococcal surface proteins can support biofilm formation. Representative isolates from the major lineages of MRSA form protein-dependent biofilm *in vitro* suggesting that this is likely to be of medical relevance (O'Neill et al., 2007). The importance of FnBP- and Aap-dependent biofilm formation has been demonstrated *in vivo* using animal models of foreign body infection (Vergara-Irigaray et al., 2009; Schaeffer et al., 2014). It will be important to determine how widespread FnBP-mediated biofilm formation is among *S. aureus* strains from different genetic backgrounds. Biofilm formation by certain isolates of HA-MRSA from CC8 and CC22 and CA-MRSA of the USA300 lineage (CC8) is dependent on FnBPs (O'Neill et al., 2008; McCourt et al., 2014). Studies to assess the contribution of FnBPs to biofilm formation should be extended to all major classes of CA- and HA-MRSA. It will also be important to establish if other surface proteins contribute to biofilm accumulation in clinically relevant strains and to study their role using animal models of infection.

Further insights into the mechanistic basis of surface protein-mediated biofilm formation will inform the design of specific inhibitors of the protein–protein interactions involved in biofilm accumulation. Small molecules or peptides could be used to prevent or treat biofilm-associated infection.

### Conflict of interest statement

The authors declare that the research was conducted in the absence of any commercial or financial relationships that could be construed as a potential conflict of interest.

## References

[B1] Abraham N. M. Jefferson K. K. (2012). *Staphylococcus aureus* clumping factor B mediates biofilm formation in the absence of calcium. Microbiology 158, 1504–1512. 10.1099/mic.0.057018-0 22442307 PMC3541775

[B2] Arrizubieta M. J. Toledo-Arana A. Amorena B. Penades J. R. Lasa I. (2004). Calcium inhibits bap-dependent multicellular behavior in *Staphylococcus aureus*. J. Bacteriol. 186, 7490–7498. 10.1128/JB.186.22.7490-7498.2004 15516560 PMC524893

[B3] Barbu E. M. Mackenzie C. Foster T. J. Höök M. (2014). SdrC induces staphylococcal biofilm formation through a homophilic interaction. Mol. Microbiol. 94, 172–185. 10.1111/mmi.12750 25115812 PMC5718044

[B4] Beenken K. E. Spencer H. Griffin L. M. Smeltzer M. S. (2012). Impact of extracellular nuclease production on the biofilm phenotype of *Staphylococcus aureus* under Calcium inhibits bap and *in vivo* conditions. Infect. Immun. 80, 1634–1638. 10.1128/IAI.06134-11 22354028 PMC3347440

[B5] Brady R. A. O'May G. A. Leid J. G. Prior M. L. Costerton J. W. Shirtliff M. E. (2011). Resolution of *Staphylococcus aureus* biofilm infection using vaccination and antibiotic treatment. Infect. Immun. 79, 1797–1803. 10.1128/IAI.00451-10 21220484 PMC3067561

[B6] Boles B. R. Horswill A. R. (2008). Agr-mediated dispersal of *Staphylococcus aureus* biofilms. PLoS Pathog. 4:e1000052. 10.1371/journal.ppat.1000052 18437240 PMC2329812

[B7] Bose J. L. Lehman M. K. Fey P. D. Bayles K. W. (2012). Contribution of the *Staphylococcus aureus* Atl AM and GL murein hydrolase activities in cell division, autolysis, and biofilm formation. PLoS ONE 7:e42244. 10.1371/journal.pone.0042244 22860095 PMC3409170

[B8] Caiazza N. C. O'Toole G. A. (2003). Alpha-toxin is required for biofilm formation by *Staphylococcus aureus*. J. Bacteriol. 185, 3214–3217. 10.1128/JB.185.10.3214-3217.2003 12730182 PMC154062

[B9] Chavakis T. Wiechmann K. Preissner K. T. Herrmann M. (2005). *Staphylococcus aureus* interactions with the endothelium: the role of bacterial “secretable expanded repertoire adhesive molecules” (SERAM) in disturbing host defense systems. Thromb. Haemost. 94, 278–285. 10.1160/TH05-05-0306 16113816

[B10] Christner M. Franke G. C. Schommer N. N. Wendt U. Wegert K. Pehle P.. (2010). The giant extracellular matrix-binding protein of *Staphylococcus epidermidis* mediates biofilm accumulation and attachment to fibronectin. Mol. Microbiol. 75, 187–207. 10.1111/j.1365-2958.2009.06981.x 19943904

[B11] Conlon B. P. Geoghegan J. A. Waters E. M. McCarthy H. Rowe S. E. Davies J. R.. (2014). A role for the A-domain of unprocessed accumulation associated protein (Aap) in the attachment phase of the Staphylococcus epidermidis biofilm phenotype. J. Bacteriol. 196, 4268–4275. 10.1128/JB.01946-14 25266380 PMC4248850

[B12] Conrady D. G. Brescia C. C. Horii K. Weiss A. A. Hassett D. J. Herr A. B. (2008). A zinc-dependent adhesion module is responsible for intercellular adhesion in staphylococcal biofilms. Proc. Natl. Acad. Sci. U.S.A. 105, 19456–19461. 10.1073/pnas.0807717105 19047636 PMC2592360

[B13] Conrady D. G. Wilson J. J. Herr A. B. (2013). Structural basis for Zn^2+^-dependent intercellular adhesion in staphylococcal biofilms. Proc. Natl. Acad. Sci. U.S.A. 110, E202–E211. 10.1073/pnas.1208134110 23277549 PMC3549106

[B13a] Corrigan R. M. Rigby D. Handley P. Foster T. J. (2007). The role of Staphylococcus aureus surface protein SasG in adherence and biofilm formation. Microbiology 153, 2435–2446. 10.1099/mic.0.2007/006676-0 17660408

[B14] Cucarella C. Solano C. Valle J. Amorena B. Lasa I. Penades J. R. (2001). Bap, a *Staphylococcus aureus* surface protein involved in biofilm formation. J. Bacteriol. 183, 2888–2896. 10.1128/JB.183.9.2888-2896.2001 11292810 PMC99507

[B15] Foulston L. Elsholz A. K. DeFrancesco A. S. Losick R. (2014). The extracellular matrix of *Staphylococcus aureus* biofilms comprises cytoplasmic proteins that associate with the cell surface in response to decreasing pH. MBio 5, e01667–e01614. 10.1128/mBio.01667-14 25182325 PMC4173787

[B16] Foster T. J. Geoghegan J. A. Ganesh V. K. Hook M. (2014). Adhesion, invasion and evasion: the many functions of the surface proteins of *Staphylococcus aureus*. Nat. Rev. Microbiol. 12, 49–62. 10.1038/nrmicro3161 24336184 PMC5708296

[B17] Fowler V. G. Sakoulas G. McIntyre L. M. Meka V. G. Arbeit R. D. Cabell C. H.. (2004). Persistent bacteremia due to methicillin-resistant *Staphylococcus aureus* infection is associated with *agr* dysfunction and low-level *in vitro* resistance to thrombin-induced platelet microbicidal protein. J. Infect. Dis. 190, 1140–1149. 10.1086/423145 15319865

[B18] Geoghegan J. A. Corrigan R. M. Gruszka D. T. Speziale P. O'Gara J. P. Potts J. R.. (2010). Role of surface protein SasG in biofilm formation by *Staphylococcus aureus*. J. Bacteriol. 192, 5663–5673. 10.1128/JB.00628-10 20817770 PMC2953683

[B19] Geoghegan J. A. Monk I. R. O'Gara J. P. Foster T. J. (2013). Subdomains N2N3 of fibronectin binding protein A mediate *Staphylococcus aureus* biofilm formation and adherence to fibrinogen using distinct mechanisms. J. Bacteriol. 195, 2675–2683. 10.1128/JB.02128-12 23564165 PMC3676058

[B20] Gil C. Solano C. Burgui S. Latasa C. García B. Toledo-Arana A.. (2014). Biofilm matrix exoproteins induce a protective immune response against *Staphylococcus aureus* biofilm infection. Infect. Immun. 82, 1017–1029. 10.1128/IAI.01419-13 24343648 PMC3957983

[B21] Gross M. Cramton S. E. Gotz F. Peschel A. (2001). Key role of teichoic acid net charge in *Staphylococcus aureus* colonization of artificial surfaces. Infect. Immun. 69, 3423–3426. 10.1128/IAI.69.5.3423-3426.2001 11292767 PMC98303

[B22] Gruszka D. T. Wojdyla J. A. Bingham R. J. Turkenburg J. P. Manfield I. W. Steward A.. (2012). Staphylococcal biofilm-forming protein has a contiguous rod-like structure. Proc. Natl. Acad. Sci. U.S.A. 109, E1011–E1018. 10.1073/pnas.1119456109 22493247 PMC3340054

[B23] Hammer N. P. Skaar E. P. (2011). Molecular mechanisms of *Staphylococcus aureus* iron acquisition. Annu. Rev. Microbiol. 65, 129–147. 10.1146/annurev-micro-090110-102851 21639791 PMC3807827

[B24] Houston P. Rowe S. E. Pozzi C. Waters E. M. O'Gara J. P. (2011). Essential role for the major autolysin in the fibronectin-binding protein-mediated *Staphylococcus aureus* biofilm phenotype. Infect. Immun. 79, 1153–1165. 10.1128/IAI.00364-10 21189325 PMC3067512

[B25] Hu J. Xu T. Zhu T. Lou Q. Wang X. Wu Y.. (2011). Monoclonal antibodies against accumulation-associated protein affect EPS biosynthesis and enhance bacterial accumulation of *Staphylococcus epidermidis*. PLoS ONE 6:e20918. 10.1371/journal.pone.0020918 21687690 PMC3110253

[B26] Huseby M. J. Kruse A. C. Digre J. Kohler P. L. Vocke J. A. Mann E. E.. (2010). Beta toxin catalyzes formation of nucleoprotein matrix in staphylococcal biofilms. Proc. Natl. Acad. Sci. U.S.A. 107, 14407–14412. 10.1073/pnas.0911032107 20660751 PMC2922554

[B27] Johnson M. Cockayne A. Morrissey J. A. (2008). Iron-regulated biofilm formation in *Staphylococcus aureus* Newman requires *ica* and the secreted protein Emp. Infect. Immun. 76, 1756–1765. 10.1128/IAI.01635-07 18268030 PMC2292859

[B28] Kiedrowski M. R. Kavanaugh J. S. Malone C. L. Mootz J. M. Voyich J. M. Smeltzer M. S.. (2011). Nuclease modulates biofilm formation in community-associated methicillin-resistant *Staphylococcus aureus*. PLoS ONE 6:e26714. 10.1371/journal.pone.0026714 22096493 PMC3214024

[B29] Lam H. Kesselly A. Stegalkina S. Kleanthous H. Yethon J. A. (2014). Antibodies to PhnD inhibit staphylococcal biofilms. Infect. Immun. 82, 3764–3774. 10.1128/IAI.02168-14 24958708 PMC4187816

[B30] Li M. Du X. Villaruz A. E. Diep B. A. Wang D. Song Y.. (2012). MRSA epidemic linked to a quickly spreading colonization and virulence determinant. Nat. Med. 18, 816–819. 10.1038/nm.2692 22522561 PMC3378817

[B30a] Mack D. Fischer W. Krokotsch A. Leopold K. Hartmann R. Egge H.. (1996). The intercellular adhesin involved in biofilm accumulation of Staphylococcus epidermidis is a linear beta-1,6-linked glucosaminoglycan: purification and structural analysis. J. Bacteriol. 178, 175–183. 8550413 10.1128/jb.178.1.175-183.1996 PMC177636

[B31] McCourt J. O'Halloran D. P. McCarthy H. O'Gara J. P. Geoghegan J. A. (2014). Fibronectin-binding proteins are required for biofilm formation by community-associated methicillin-resistant *Staphylococcus aureus* strain LAC. FEMS Microbiol. Lett. 353, 157–164. 10.1111/1574-6968.12424 24628034

[B32] Merino N. Toledo-Arana A. Vergara-Irigaray M. Valle J. Solano C. Calvo E.. (2009). Protein A-mediated multicellular behavior in *Staphylococcus aureus*. J. Bacteriol. 191, 832–43. 10.1128/JB.01222-0 19047354 PMC2632097

[B33] Missineo A. Di Poto A. Geoghegan J. A. Rindi S. Heilbronner S. Gianotti V.. (2014). IsdC from *Staphylococcus lugdunensis* induces biofilm formation under low-iron growth conditions. Infect. Immun. 82, 2448–2459. 10.1128/IAI.01542-14 24686057 PMC4019187

[B34] Montanaro L. Poggi A. Visai L. Ravaioli S. Campoccia D. Speziale P.. (2011). Extracellular DNA in biofilms. Int. J. Artif. Organs. 34, 824–831. 10.5301/ijao.5000051 22094562

[B35] O'Neill E. Pozzi C. Houston P. Humphreys H. Robinson D. A. Loughman A.. (2008). A novel *Staphylococcus aureus* biofilm phenotype mediated by the fibronectin-binding proteins, FnBPA and FnBPB. J. Bacteriol. 190, 3835–3850. 10.1128/JB.00167-08 18375547 PMC2395027

[B36] O'Neill E. Pozzi C. Houston P. Smyth D. Humphreys H. Robinson D. A.. (2007). Association between methicillin susceptibility and biofilm regulation in *Staphylococcus aureus* isolates from device-related infections. J. Clin. Microbiol. 45, 1379–1388. 17329452 10.1128/JCM.02280-06 PMC1865887

[B37] O'Toole G. Kaplan H. B. Kolter R. (2000). Biofilm formation as microbial development. Annu. Rev. Microbiol. 54, 49–79. 10.1146/annurev.micro.54.1.49 11018124

[B38] Otto M. (2013). *Staphylococcal* infections: mechanisms of biofilm maturation and detachment as critical determinants of pathogenicity. Annu. Rev. Med. 64, 175–188. 10.1146/annurev-med-042711-140023 22906361

[B39] Periasamy S. Joo H. S. Duong A. C. Bach T. H. Tan V. Y. Chatterjee S. S.. (2012). How *Staphylococcus aureus* biofilms develop their characteristic structure. Proc. Natl. Acad. Sci. U.S.A. 109, 1281–1286. 10.1073/pnas.1115006109 22232686 PMC3268330

[B40] Resch A. Rosenstein R. Nerz C. Götz F. (2005). Differential gene expression profiling of *Staphylococcus aureus* cultivated under biofilm and planktonic conditions. Appl. Environ. Microbiol. 71, 2663–2676. 10.1128/AEM.71.5.2663-2676.2005 15870358 PMC1087559

[B41] Roche F. M. Meehan M. Foster T. J. (2003). The *Staphylococcus aureus* surface protein SasG and its homologues promote bacterial adherence to human desquamated nasal epithelial cells. Microbiology 149, 2759-2567. 10.1099/mic.0.26412-0 14523109

[B42] Rohde H. Burdelski C. Bartscht K. Hussain M. Buck F. Horstkotte M. A.. (2005). Induction of *Staphylococcus epidermidis* biofilm formation via proteolytic processing of the accumulation-associated protein by staphylococcal and host proteases. Mol. Microbiol. 55, 1883–1895. 10.1111/j.1365-2958.2005.04515.x 15752207

[B43] Rudkin J. K. Edwards A. M. Bowden M. G. Brown E. L. Pozzi C. Waters E. M.. (2012). Methicillin resistance reduces the virulence of healthcare-associated methicillin-resistant *Staphylococcus aureus* by interfering with the *agr* quorum sensing system. J. Infect. Dis. 205, 798–806. 10.1093/infdis/jir845 22301683 PMC3318674

[B44] Rupp M. E. Fey P. D. Heilmann C. Götz F. (2001). Characterization of the importance of *Staphylococcus epidermidis* autolysin and polysaccharide intercellular adhesin in the pathogenesis of intravascular catheter-associated infection in a rat model. J. Infect. Dis. 183, 1038–1042. 10.1086/319279 11237828

[B45] Sanchez C. J. Shivshankar P. Stol K. Trakhtenbroit S. Sullam P. M. Sauer K.. (2010). The pneumococcal serine-rich repeat protein is an intra-species bacterial adhesin that promotes bacterial aggregation *in vivo* and in biofilms. PLoS Pathog. 6:e1001044. 10.1371/journal.ppat.1001044 20714350 PMC2920850

[B46] Schaeffer C. R. Woods K. M. Longo G. M. Kiedrowski M. R. Paharik A. E. Büttner H.. (2014). Accumulation-associated protein (Aap) enhances *Staphylococcus epidermidis* biofilm formation under dynamic conditions and is required for infection in a rat catheter model. Infect Immun. [Epub ahead of print]. 10.1128/IAI.02177-14 25332125 PMC4288872

[B47] Schroeder K. Jularic M. Horsburgh S. M. Hirschhausen N. Neumann C. Bertling A.. (2009). Molecular characterization of a novel *Staphylococcus aureus* surface protein (SasC) involved in cell aggregation and biofilm accumulation. PLoS ONE 4:e7567. 10.1371/journal.pone.0007567 19851500 PMC2761602

[B48] Schwartz K. Sekedat M. D. Syed A. K. O'Hara B. Payne D. E. Lamb A.. (2014). The AgrD N-terminal leader peptide of *Staphylococcus aureus* has cytolytic and amyloidogenic properties. Infect. Immun. 82, 3837–3844. 10.1128/IAI.02111-14 24980969 PMC4187843

[B49] Schwartz K. Syed A. K. Stephenson R. E. Rickard A. H. Boles B. R. (2012). Functional amyloids composed of phenol soluble modulins stabilize *Staphylococcus aureus* biofilms. PLoS Pathog. 8:e1002744. 10.1371/journal.ppat.1002744 22685403 PMC3369951

[B50] Shahrooei M. Hira V. Khodaparast L. Khodaparast L. Stijlemans B. Kucharíková S.. (2012). Vaccination with SesC decreases *Staphylococcus epidermidis* biofilm formation. Infect. Immun. 80, 3660–3668. 10.1128/IAI.00104-12 22802343 PMC3457580

[B51] Sharma-Kuinkel B. K. Mann E. E. Ahn J. S. Kuechenmeister L. J. Dunman P. M. Bayles K. W. (2009). The *Staphylococcus aureus* LytSR two-component regulatory system affects biofilm formation. J. Bacteriol. 191, 4767–4775. 10.1128/JB.00348-09 19502411 PMC2715716

[B52] Vaudaux P. E. François P. Proctor R. A. McDevitt D. Foster T. J. Albrecht R. M.. (1995). Use of adhesion-defective mutants of *Staphylococcus aureus* to define the role of specific plasma proteins in promoting bacterial adhesion to canine arteriovenous shunts. Infect. Immun. 63, 585–590. 7822026 10.1128/iai.63.2.585-590.1995 PMC173036

[B53] Vergara-Irigaray M. Valle J. Merino N. Latasa C. García B. Ruiz de Los Mozos I.. (2009). Relevant role of fibronectin-binding proteins in *Staphylococcus aureus* biofilm-associated foreign-body infections. Infect. Immun. 77, 3978–3991. 10.1128/IAI.00616-09 19581398 PMC2738049

[B54] Wang R. Khan B. A. Cheung G. Y. Bach T. H. Jameson-Lee M. Kong K. F.. (2011). *Staphylococcus epidermidis* surfactant peptides promote biofilm maturation and dissemination of biofilm-associated infection in mice. J. Clin. Invest. 121, 238–248. 10.1172/JCI42520 21135501 PMC3007140

[B55] Yang Y. H. Jiang Y. L. Zhang J. Wang L. Bai X. H. Zhang S. J.. (2014). Structural insights into SraP-mediated *Staphylococcus aureus* adhesion to host cells. PLoS Pathog. 10:e1004169. 10.1371/journal.ppat.1004169 24901708 PMC4047093

